# Heterogeneity of Capability Deprivation and Subjective Sense of Gain: Analysis of Factor Mixture Models Based on 892 Rural Households in Six Provinces

**DOI:** 10.3390/ijerph19074294

**Published:** 2022-04-03

**Authors:** Zenghui Huo, Mei Zhang, Junhui Han

**Affiliations:** 1 College of Economics & Management, China Jiliang University, Hangzhou 310018, China; huozenghui@cjlu.edu.cn; 2 College of Economics and Management, Zhejiang University of Water Resources and Electric Power, Hangzhou 310018, China; 3 School of Economics and Management, Taiyuan University of Technology, Taiyuan 030024, China; hanjunhui@tyut.edu.cn

**Keywords:** capability approach, functionings and opportunities, differences between groups, subjective sense of gain, association relationship

## Abstract

Background: The capability approach conceptualizes poverty as capability deprivation. Given that functionings and opportunities as key factors are diverse, the combination of a varied lack of functionings and opportunities will lead to different deprivation patterns. Therefore, we sought to investigate the association between the category of capacity deprivation and the subjective acquisition of rural households. Methods: Data were collected from the micro survey of 892 households in six provinces. The overall sense of acquisition was measured by self-assessment of life satisfaction and the relative sense of gain was measured by self-assessment of social status and communication confidence. Capability deprivation was assessed through evaluation functionings and opportunities for a better life, such as education, social security, medical and health services, living conditions and spiritual and cultural activities. The factor mixture models were used to investigate the group categories of capability deprivation and ordered probit regression was used to estimate the associations between the categories of capability deprivation and sense of gain. Results: There were mild, moderate and severe differences among the deprivation items reflecting functionings and opportunities. In addition, capability deprivation was grouped into four classes: double deprivation of functionings and opportunities, severe deprivation of opportunities, functionings deprivation and non-poverty. There was a significant negative relationship between deprivation classes and the subjective sense of gain. Conclusion: The deprivation from some social participation functions and services led to a reduced sense of acquisition. However, serious deprivations from educational opportunities and social participation opportunities were the main reason for the lower sense of gain. Eliminating the unequal educational opportunities and social participation opportunities for people is imperative to improving the subjective sense of gain.

## 1. Introduction

In 2020, the Chinese government announced that all rural residents living below the existing poverty line were to be lifted out of poverty. Chinese rural poor people were free from worries over food and clothing and had access to compulsory education, basic medical services and safe housing. The existing poverty standard helped to solve the problem of meeting people’s basic living needs. However, in the new stage of development, the principal contradiction facing the Chinese society has changed into one between unbalanced and inadequate development and the people’s ever-growing needs for a better life. In keeping with the changes in the major social problems, the CPC Central Committee has proposed that China’s poverty alleviation strategy should now focus on solving relative poverty. Therefore, there is a need to explore the establishment of a long-term mechanism to solve relative poverty with the view to enhancing the people’s sense of gain. Relative poverty refers to relative deprivation in terms of the average living standard of the society, including the lack of basic survival needs, life needs and power needs [1]. At present, the people’s needs for a better life should be manifested in the pursuit of developmental needs such as education, medical care, employment and social security [2]. However, the mismatch between the lack of feasible ability caused by unbalanced and inadequate development and needs for a better life will lead to the ability deprivation of vulnerable groups. Sen (1985) believed that poverty was essentially the deprivation of people’s feasible ability, while feasible ability was the substantial freedom to realize various possible combinations of functional activities [3]. The capability approach transferred poverty identification from resource space to functional space and conceptualized poverty as capacity deprivation. The intrinsic importance lies in the functional and capability elements in the functional space [4]. Due to the unrealized diversity of functions and opportunities in the assessment space, different combinations of functions and opportunities could constitute different deprivation patterns. Exploring the differences of deprivation patterns is helpful to accurately identify the relatively poor.

The Fifth Plenary Session of the 19th CPC Central Committee adopted the Proposal, which emphasized improving the quality of people’s lives, making solid efforts to promote common prosperity and constantly enhancing the people’s sense of gain; they formulated the 14th Five-Year Plan for National Economic and Social Development and the Long-term Goals for 2035. In contrast to the sense of happiness, the sense of gain emphasizes the real acquisition of people’s livelihood and is considered as the people’s understanding of the realization status and degree of interests at different levels such as material, spiritual and rights [5]. The literature has paid extensive attention to these issues, measuring residents’ sense of gain [6], examining the impact of social policies on residents’ sense of gain [7,8,9], and examining the impact of micro-socio-economic status [10,11], outside employment [12,13], community support [14] and social equity [15] on residents’ sense of gain. In addition, the correlation between relative poverty and residents’ sense of gain is also considered. In terms of relevance, relative poverty can be regarded as a social psychological problem to some extent and it is based on the evaluation of subjective feelings of gain, security and happiness [16]. From the perspective of causality, income relative poverty and subjective relative poverty will significantly reduce the happiness level of farmers [17]. Ability deprivation can also significantly reduce residents’ life satisfaction [18]. The study aimed to investigate the relationship between the categories of capability deprivation and the sense of gain in households in rural China by using the capability approach. Based on the reality of Chinese rural households’ needs for a better life, this study used the Factor Mixture Models to evaluate the internal pattern differences of capability deprivation. The study further investigates the impact of the pattern differences of capability deprivation on rural households’ sense of gain by using ordered probit regression.

## 2. Literature Review

### 2.1. Capability Deprivation and Relative Poverty of Rural Households in China

Townsend (1979) proposed the theory of relative poverty and considered that poverty was the relative deprivation of ‘being excluded from normal lifestyles, customs and social activities due to the lack of control over adequate resources’ [19]. In addition to the lack of controllable resources of the family, the causes of deprivation also included the loss of human rights and the lack of capacity. In the same vein, Sen (1985) proposed the Capability Approach (CA), arguing that poverty is manifested as low quality of life which is rooted in the insufficient ability to transform resources into activities and states [3]. The capability method emphasized the capacity of different individuals and groups in society to depend on each other and provided a theoretical basis for the understanding ability, social exclusion and relative poverty between concepts [20]. The capacity approach has been widely used in poverty measurement. The Human Poverty Index (HPI) and Multidimensional Poverty Index (MPI) developed by the United Nations are the most important examples. Alkire and Foster (2011) proposed a double cutoff method to measure Multidimensional Poverty Index; it assumes that the weights of all dimensions are equal [21]. Recent studies show that the functional dimension has been extended. When the World Bank reports on global multidimensional poverty, it extends the functional dimension to other aspects such as inadequate income and family security [22]. Multidimensional poverty studies in Europe include material deprivation and employment deprivation [23,24,25]. At the same time, opportunity deprivation has attracted some attention. Anand et al., (2020) developed direct ability indicators such as family life, work opportunities, social participation opportunities and service opportunities to investigate multidimensional poverty in the United States and the United Kingdom [26].

Previously, Chinese researchers introduced multidimensional poverty indicators to investigate the absolute poverty of rural households. Wang et al., (2009) used the Alkire–Foster method to measure multidimensional poverty of rural households in China [27]. Zou et al., (2011) investigated the dynamic changes of multidimensional poverty [28], Zhou et al., (2017) investigated the long-term multidimensional poverty of families [29] and Zhu et al., (2017) used the multidimensional poverty method to test the targeting rate of low-income families [30]. In their research, the A–F double cutoff method was widely used and the weights of each dimension were set to be equal. Recent studies show that the multidimensional poverty framework is being used in the study of relative poverty. Wang et al., (2020) believed that China’s relative poverty should be measured by multidimensional standards after 2020 [31]. Wang et al., (2021) adopted multidimensional criteria including income and non-income to investigate the multidimensional relative poverty differences between urban and rural residents based on the data of the China Household Survey in 2018 [2].

### 2.2. The Relationship between Capability Deprivation and Subjective Acquisition

The relationship between competence and subjective well-being is an important issue in poverty research. First, is the relationship between economic conditions and subjective well-being. Easterlin (1995) believed that an increase in average income generally did not significantly improve life satisfaction [32]. Similarly, Kingdon et al., (2006) found that relative income levels reduced personal happiness [33]. In addition, Clark et al., (2016) found that the incidence and depth of income poverty would reduce life satisfaction [34]. In particular, Samman et al., (2013) found that when the poor were still below the poverty line, the increase of income did not significantly affect their satisfaction [35]. Second, is the relationship between ability deprivation and subjective well-being. Deprivation explained changes in life satisfaction more than income poverty did. People who suffered from material and social deprivation had lower life satisfaction and quality of life [36,37,38].

In China, ‘what factors affect residents’ subjective sense of gain’ is attracting widespread attention. Firstly, social policy is an important factor in residents’ sense of gain. Cheng (2021) found that the reform of exempting agricultural tax relieved farmers’ burden, narrowed the income gap between urban and rural areas and thus improved farmers’ sense of gain [7]. In addition, Ye et al., (2021) believed that participating in the new rural insurance significantly improved the overall sense of economic gain of the elderly from poor families [8]. Meanwhile, Xu et al., (2021) found that social quality such as social economic security, social cohesion and social empowerment can significantly improve urban residents’ sense of gain [9]. Secondly, social and economic status, professional identity and other personal characteristics are also important factors. Wang et al., (2018) found that socio-economic status had a significant positive impact on the sense of acquisition [6]. In the same vein, Xu et al., (2021) found that objective socio-economic status and residential space affected people’s sense of acquisition through their subjective socio-economic status [10]. In addition, Wu et al., (2021) found that the employment of migrant workers not only improved farmers’ income, but also improved their subjective feelings on income level and life satisfaction [12]. Finally, community identity and social capital have mediating effects. For instance, Wang et al., (2020) found that community identity mediated the relationship between socio-economic status and sense of gain. Promoting the mobility of socio-economic status and actively intervening in community identity is conducive for improving the sense of gain [14]. Liu et al., (2020) found that there is a significantly negative mediating effect of linking social capital with a negative association between subjective relative deprivation and life satisfaction [39].

### 2.3. Summary of Literature

To sum up, multidimensional deprivation and subjective sense of gain are becoming an important perspective of relative poverty management in China. However, from the perspective of research, most literature focused on the functional level of poverty and lacked investigation on opportunity deprivation. In particular, there is limited literature on how deprivation affects rural households’ sense of gain. In addition, as far as the measurement method is concerned, the A–F double cutoff method usually assumes the same weight of all dimensions, so it is difficult to reflect the difference of deprivation degree of different groups with the same deprivation threshold. As Ravallion (2012) pointed out, multidimensional poverty results generated by the ‘mashup’ aggregation method created worrying doubts about their policy relevance due to insufficient attention to the uncertainty of their constituent indicators and weights [40]. In view of this, based on the key concepts of function and capacity in the capacity framework, the index system of Chinese rural households’ capacity deprivation was constructed. The factor mixture model based on the objective characteristics of the data was used to endogenously classify the samples. On this basis, the influence of different ability deprivation groups on rural households’ subjective acquisition was analyzed.

## 3. Data and Variable Explanation

### 3.1. Data Sources

In this article, the stratified sampling method was adopted. The investigation team carried out field household surveys in Zhejiang, Jiangxi, Hubei, Hebei, Yunnan and Guizhou provinces from July to August 2020. Considering the degree of economic development of each province, 5–6 villages were selected as the sample villages, and 20–25 households were investigated in each village. Finally, 34 villages were investigated, and 892 valid questionnaires were obtained, with an effective rate of 97.27%. The questionnaire mainly involves the demographic information of the respondents, their answers to questions related to the better life as well as their family income composition and demographic structure.

### 3.2. Measures of Subjective Sense of Acquisition

The subjective sense of acquisition is defined as people’s positive psychological experience and subjective satisfaction with the achievements of China’s reform and opening up. According to the reference group theory, people are more inclined to compare with similar people due to the lack of objective standards when evaluating their opinions and abilities [41]. For farmers, groups in the same village not only have the same social structure with each other, but also are more familiar with each other or have easier access to information [42]. Therefore, other farmers in the same village were used as the reference objects for horizontal comparison and ‘self–assessment of social status in village’ and ‘self–assessment of communication confidence’ were used as the observable indicators of relative sense of gain. At the same time, ‘self–assessment of life satisfaction’ was taken as the observable index of overall acquisition. Thus, a self-reporting method was adopted to measure sense of acquisition. The responses ‘very dissatisfied’, ‘relatively dissatisfied’, ‘satisfied’, ‘relatively satisfied’ and ‘very satisfied’ were assigned a value of 1–5, respectively. The descriptive statistics of sense of acquisition are shown in Table 1.

### 3.3. Measures of Capability Deprivation

The capability approach is a broad normative framework for assessing individual well-being, social arrangements, policy design and recommendations for social change, with the core proposition being an assessment of a person’s quality of life [43]. From the perspective of activities, experience and ability, poverty means low quality of life [3]. We consider that in the Chinese scenario, rural households’ ability deprivation is a mismatch between the quality of life and the feasible ability to achieve a better life. Core concepts include resources, transformation factors, capabilities and functions. Function is the existence and state of life that people have reason to cherish, emphasizing the achievement of happiness; it includes not only basic functions such as good nutrition, good shelter and health, but also complex functions such as social participation, self-esteem, happiness and safety [25]. Ability is the opportunity to achieve a valuable function, emphasizing the freedom of happiness. From the perspective of formation mechanism, income, goods, services and other resources as input, through personal factors, social factors, environmental factors and other conversion factors resources are converted into capabilities and then capabilities are used to achieve functions. Poverty is the result of deprivation of capacity; the combination of all the functions available to an individual. Functional deprivation and opportunity deprivation are important perspectives for examining multidimensional poverty [44]. They are built on the combination of daily goods, activities and services, because they have specific and valuable functions. This article examines the aspects of education, social security, medical and health services, living conditions and spiritual and cultural life of the good life. Table 2 lists information about life activities, services and the specific functions and opportunities involved.

Better education, more reliable social security and better medical and health services are important components of a better life for the people. A better life in education is reflected by designing ‘a desirable primary school nearby’ or ‘a desirable secondary school nearby’, among others. They can capture the opportunity to pursue knowledge, thinking and reasoning ability. Through the design of ‘buying endowment insurance’, ‘giving alimony to elders’, ‘emergency deposit’, etc., to reflect the better life of social security, they can capture social ‘shelter’, security and other complex functions. Through the design of ‘serious illness to urban hospital’, ‘paying for large medical expenses’ and ‘annual physical examination’ they can reflect the better life of medical and health services. They can capture the basic function of avoiding serious incurable illness and obtain the basic disease prevention machine.

Comfortable living conditions and rich spiritual and cultural life are also part of people’s better life. By designing ‘to courier service’, ‘banking services’ and ‘garbage sorting service’ to reflect the better life in terms of living conditions, they can capture rural households’ opportunities to enjoy convenient living service conditions. Through the design of ‘culture to the countryside’, ‘travel’, ‘neighbors helping each other’ and other aspects of habitual social activities reflecting the better life, they can capture the sense of the belonging function or the opportunity of rural households’ to participate in social activities. The index can capture the function or opportunity for rural households to participate in social activities in an intimate way by designing ‘holiday dinners out’, ‘exchanging gifts and money in daily life’ and ‘giving red envelopes during the Spring Festival’ to reflect the better life of traditional culture.

## 4. Methods

The counting method and the A–F method are usually used to measure the severity of multidimensional deprivation, but their disadvantage is that they cannot identify the group difference of the same deprivation score. Due to the complexity of multiple deprivation combinations, the deprivation group difference pattern cannot be observed directly, but it can be regarded as a latent category variable. The latent class analysis (LCA) estimates latent categories through different joint probabilities of individuals on observational indicators; however, its statistical limitation lies in the assumption of local independence, which is often difficult to achieve [45]. Therefore, this article adopts the factor mixing mode that relaxes the assumption of local independence, which includes the following three parts.

### 4.1. Factor Analysis

The purpose of factor analysis is to explore the latent continuous variables behind the subject items, i.e., the common factors. It is assumed that all individuals in the sample come from the same homogenous population and different factor variations lead to differences among individuals. For the *i*th individual, the factor analysis model of the response Y of the binary test item is:

(1) $$y_{i}{}^{*}=\Lambda \eta_{i}+\varepsilon_{i}$$

(2) $$\eta_{i}=\alpha +\xi_{i}$$

(3) $$y_{ij}=1\;if\;y_{ij}^{*}>\tau_{j}\;and\;y_{ij}^{*}=0\;if\;y_{ij}{}^{*}\leq \;\tau_{j}\;$$

$y_{i}\;$is the response of individual i, $y_{i}{}^{*}$ is the potential response vector of individual and $\Lambda_{k}$ is factor loading. η is the factor vector, α is the factor mean vector, $\xi_{it}$ is a normally distributed factor residual matrix with a mean of 0 and a variance of $\Psi_{t}$ and $\tau_{j}$ is an item threshold vector.

Since capacity deprivation is considered a unidimensional latent variable, there is only one common factor. Therefore, the article adopts the two-parameter IRT generalized latent response model:

(4) $$y_{ij}^{*}=\beta_{j}+\lambda_{j}\eta_{i}+\varepsilon_{i}$$

(5) $$y_{ij}=1\;if\;y_{ij}^{*}>0\;and\;y_{ij}^{*}=0\;if\;y_{ij}{}^{*}\leq \;0$$

For a particular response to a potential trait or factor, the conditional probability is linked by logarithmic probability. Item parameters β and λ were obtained by maximum likelihood estimation and the predicted values of potential traits were estimated by the empirical Bayes’ method. Factor loading λ is called differentiation parameter and the intercept β is called the degree of difficulty.

### 4.2. Latent Class Model

Factor analysis focuses on the classification of test items, while latent class analysis focuses on the classification of subjects or individuals [46]. Local independence is an important assumption of the latent class model. The association of any two observation indicators in a category has been explained by latent class variables and they are independent of each other. The basic function of the latent class model is in the form:

(6) $$p\left(y_{j}\right)=\sum_{k=1}^{K}\gamma_{k}\prod_{j=1}^{J}\prod_{r_{j}}^{R_{J}}\rho_{j,r_{j}|k}{}^{I\left(y_{j}=r_{j}\right)}$$

For the latent class variable C, there are K categories (C= K, K = 1, 2… K); $\gamma_{k}$ is the latent class probability. $\rho_{j,r_{j}|k}$ reaction probability for items, under the condition of c category test item j observation response categories $r_{j}$ probability, indicates that the function $I\left(y_{j}=r_{j}\right)=1,\;if$ the response of item $r_{j}$ is j, otherwise 0. The formula shows that the probability of observing a particular response vector is a function of the latent category probability and conditional probability. On the basis of the parameter estimation and model fitting, the probability of individuals belonging to a certain category is calculated according to the Bayes’ theorem.

### 4.3. Factor Mixture Analysis (FMM)

On the basis of relaxing the assumption of local independence, the factor mixture model combines latent class analysis (LCA) and factor analysis (FA), allowing individuals to be divided into multiple categories, while allowing individual differences within each category. Individuals within a category share a common estimated parameter (factor loading) to account for individual differences within the category, but each class has a different set of parameters that can provide useful information about the severity of deprivation. For binary test item y, there is population heterogeneity among individuals, i.e., there are K categories (K = 1, 2, 3… K). FMM model is as follows [47]:

(7) $$y_{ik}=\Lambda_{k}\eta_{ik}+\varepsilon_{ik}$$

(8) $$\eta_{ik}=\alpha_{k}+\xi_{ik}$$

(9) $$\xi_{ik}\sim N\left(0,\;\;\;\Psi_{tk}\right)$$

(10) $$y_{ij}=1\;if\;y_{ij}^{*}>\tau_{j}\;and\;y_{ij}^{*}=0\;if\;y_{ij}{}^{*}\leq \;\tau_{j}\;$$

k is a latent category variable, and parameters with subscript k indicate that they can change across categories, reflecting different FMM variants. The main advantages of the factor mixture model are the new perspective of variable structure, the invariance of test measurement and the non-normal factor. The purpose of applying the factor mixture model to the study on the ability deprivation of rural households is to: (1) investigate the heterogeneity of the ability deprivation group and identify the difference of the pattern and proportion of the ability deprivation suffered by rural households, (2) factor loading can reveal the different sources of deprivation items suffered by groups with different deprivation patterns.

## 5. Empirical Results

### 5.1. Parameter Estimation of Potential Deprivation Factor

The first step is to estimate the parameters of the potential factors to reveal the parameter characteristics of deprivation of the item and to judge the effectiveness of the deprivation item. The item response model was regarded as a unidimensional CFA of classification variables and this article adopted the double parameter IRT model to estimate the parameters. Item difficulty coefficient (β) is the ability to correct answer item a 50% chance of value point location, the greater the difficulty coefficient according to serious loss of item. In addition, the discrimination coefficient (λ) reveals the changing rate of the possibility to answer correctly when ability closes to the difficulty degree. The bigger the coefficient the higher the correlation between the potential item characteristics and correct answer possibility. Before parameters are estimated, we should check the hypothesis of unidimensionality, local independence and monotonicity assumption. For this, the Stata software provides the Morgan inspection procedures for the overall judgment. When Loevinger’s H estimated value is less than 0.30, the item does not conform to the hypothesis test. Results indicated that the ‘buy endowment insurance’, ‘garbage sorting service’ and ‘travel’ and other items could not meet the hypothesis test.

**Figure 1:**
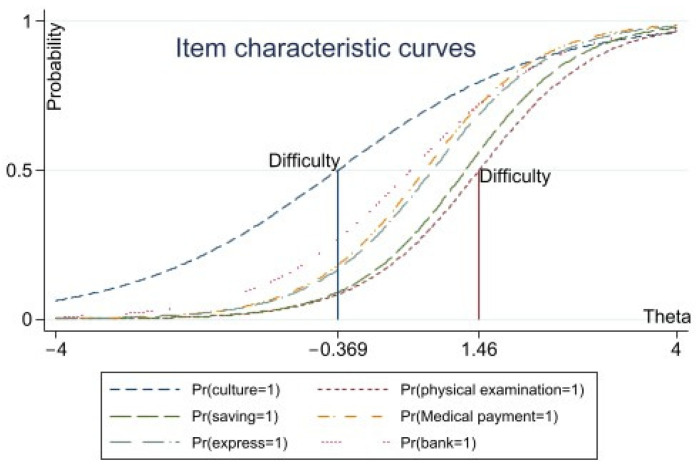
ICC of ‘moderately difficult’ items.

**Figure 2:**
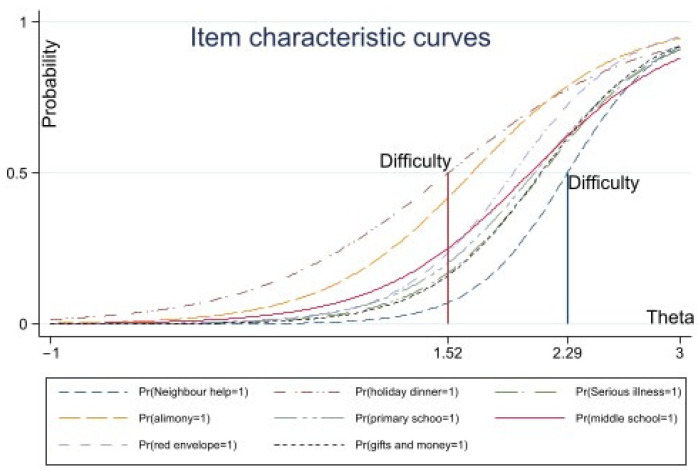
ICC of ‘higher difficult’ items.

Related research pointed out that the estimated coefficients of the discrimination coefficient and the difficulty should be neither too small nor too large; the discrimination coefficient values range was usually situated between [0, 4] [48]. If the difficulty coefficient values range was usually between [−3, 3] [49] and if the difficulty parameter was negative it manifested that more than half of the rural households were likely to suffer from the deprivation of the item. In this article, the parameter estimation results showed that the coefficients of discrimination value and difficulty coefficients were in a reasonable range. In addition, based on the difficulty coefficient, the severity of the item was classified [50]. If the difficulty coefficient was in the range of [3, 0], [0, 1.5] and [1.5, 3], respectively, the severity of the item was divided into ‘lighter’, ‘medium’ and ‘severe’. The item characteristic curve (see Figure 1 and Figure 2) shows that the item ‘culture to the countryside’ could reflect that the social participation function is the lighter deprivation, the five items ‘annual physical examination’ and ’banking services’ and others could reflect the health, security function and opportunities for service are medium deprivation and the eight items ‘attend a desirable primary school nearby’, ‘serious illness to urban hospital’ and ‘giving red envelopes for Chinese New Year’, among others, are serious deprivation.

### 5.2. Group Heterogeneity of Deprivation

The factor mix model usually needs to compare the latent class and factor of hybrid model fitting index to determine the ideal model. Model adaptation degree evaluation mainly considers information evaluation indexes such as Akaike information criterion (AIC), Bayesian information criterion (BIC), sample correction BIC (a_BIC), Entropy index and LMR test statistics. Table 3 showed that AIC and a_BIC values decreased from 7413.228 and 7456.904 of the two-class model to 7149.775 and 7236.746 of the four-class model. The Entropy index increased from 0.777 of the two-class model to 0.838 of the four-class model and this showed the accuracy of the four-class model was more than 90%. Given that the four-class model had more realistic interpretability, four classes were selected as the optimal results of rural households’ ability deprivation.

The factor mixture model was further estimated by gradually relaxing the cross-category conditions of the factor mean, factor variance, intercept, factor loading and other parameters. Although the AIC of the four-class with free intercept, variance and loading model is the smallest and the Entropy is the largest, the LMR test result is not significant. On the other hand, the four-class with free intercept and variance models have the lowest a_BIC value, significant LMR test results and a higher Entropy. Considering the interpretability of the conclusion, four-class with free intercept and variance models are selected as the ideal results.

After determining the ideal four classes, all sample farmers were divided into four subgroups: severe chance deprivation group, function deprivation group, double deprivation and non-poor group. Table 4 shows intercept estimate values of the four classes’ results. When the intercept value is −3, it means the probability of the deprived item is high. When the intercept value is 3, it means the probability of the undeprived item is low. When the intercept value is 0, it means the probability of the deprived item is 50% [51].

The first class is double deprivation of chance and ability and this class accounted for 4.6% of all samples. They are likely to have suffered from severe deprivation of social participation function such as ‘giving red envelopes for Chinese New Year’ and ‘neighbor help each other’, medium deprivation of security functions such as ‘paying for large medical expenses’, medium deprivation of service opportunities such as ‘banking services’ and ‘courier service’ and severe deprivation of severe security functions such as ‘serious illness to urban hospital’. However, they are not likely to suffer from deprivation of education chance such as ‘attend a desirable primary school nearby’ and ‘attend a desirable high school nearby’.

The second class is severe chance deprivation and this class accounted for 5.15% of all samples. They are likely to have suffered from deprivation of education chance such as ‘attend a desirable primary school nearby’ and ‘attend a desirable secondary school nearby’, deprivation of social participation function such as ‘holiday dinners out’. Additionally, they suffered from severe deprivation of social participation function such as ‘giving red envelopes for Chinese New Year’ and ‘exchanging gifts and money in daily life’. However, they are likely to have satisfactory compulsory education chance and suffer from lower deprivation of health opportunity such as ‘serious illness to urban hospital’ and lower deprivation of health function such as ‘paying for large medical expenses’.

The third class is function deprivation, and this class accounted for 22.65% of all samples. They are likely to have suffered from severe deprivation of social participation function such as ‘giving red envelopes for Chinese New Year’, ‘neighbor help each other’ and ‘exchanging gifts and money in daily life’ and medium opportunity deprivation of service opportunity such as ‘courier service’. This class is unlikely to suffer from deprivation of education opportunity, deprivation of health opportunity such as ‘serious illness to urban hospital’. The fourth class is non-poor rural households and this class accounts for 22.65% of all samples. They are likely to suffer from deprivation of public service opportunities such as ‘banking services’ and ‘courier service’ but no other deprivations.

### 5.3. Comparison of Deprivation Model with A–F Method and Income Poverty

In order to investigate the stability of the FMM results, they were first compared with the A–F double-bound method. The A–F method is a widely used multidimensional poverty measurement; it uses the weighted method to determine multidimensional deprivation scoring function and uses different thresholds K to identify multidimensional poverty [21]. It assumed that there was no difference in the multidimensional poverty degree under the same threshold value; however, there might be differences in the severity of different deprivation indicators and the multidimensional poverty severity under the same threshold level might also be different. Therefore, this article measured the subgroup proportion of FMM when the threshold K was between 0.1 and 0.5 (Table 5) to reveal the difference between the two methods. The results showed that even when K was 0.3, there were still three different deprivation patterns among multidimensional poor households. When k was raised to 0.5, it was recognized as severe multidimensional poverty. At this time, there were still two types of deprivation patterns among them. Therefore, on the basis of considering the factor loading of deprivation items, the FMM can provide more sufficient information for the overall structural differences.

Second, we also compared deprivation patterns with income poverty outcomes. Table 6 results show that the pattern of deprivation of ability was correlated with income poverty, however there were some structural differences between them. Inadequate income was still an important reason for farmers falling in capacity deprivation. The per capita income of farmers suffering from capacity deprivation was 16,575.87 Yuan, only 46.84% of the per capita income of all samples (35,388.30 Yuan) and only 37.34% of the per capita income of non-deprived farmers (44,388.11 Yuan). At the same time, the group suffering from functional deprivation was the main ‘overlap’ area of income poverty and capacity deprivation. This indicated that some indicators reflecting function and income indicators had similar functions for the identification of poor groups. On the contrary, nearly half of the households falling into relative poverty did not suffer from multiple deprivation of capacity, which reflected the different internal structures of the two poverty groups.

### 5.4. Regression Analyses

In fact, since capacity deprivation represented that farmers could not obtain some valuable functions and opportunities such as health, participation and safety, it would reduce the subjective sense of acquisition of farmers. Therefore, what were the differences in subjective acquisition among farmers subjected to different modes of deprivation? In this article, the ordered probit regression was used to investigate the correlation between ability-deprivation categories and perceived acquisition. In Table 7, models 1 to 3 provided the estimated results of the impact of different deprivation patterns on farmers’ life satisfaction, communication confidence and social status in villages, respectively. For the category variable of ability deprivation, taking non-deprived households as the reference, the Class-2 represented the subgroup in ‘severe opportunity deprivation’, the Class-3 represented the subgroup in ‘double deprivation’ and the Class-4 represented the subgroup in ‘function deprivation’. To ensure the robustness of the estimation, models 4 to 6 provided the estimated results of the impact of income poverty categories on farmers’ life satisfaction, communication confidence and social status in villages, respectively. Specifically, the income poverty lines were 40%, 50% and 60% of the median per capita income of the sample farmers, respectively. For the income poverty category variables, Poverty-1 represented households with incomes below the 40 percent poverty line, Poverty-2 represented households with incomes between 40 and 50 percent of the poverty line and Poverty-3 represented households with incomes between 50 and 60 percent of the poverty line.

Table 7 results reveal that compared with non-deprivation farmers, farmers with different types of deprivation had a lower self-evaluation of life satisfaction. This suggested that different types of valuable functional deprivation and opportunity deprivation can reduce farmers’ life satisfaction; this is consistent with findings by Suppa (2018). On the other hand, the longitudinal sense of gain such as social confidence and social status in the village might be lower for the farmers who only suffered from ‘severe opportunity deprivation’ and ‘functional deprivation’. These results indicated that some valuable social participation deprivation and educational opportunity deprivation could reduce farmers’ self-evaluation of communication confidence and social status. However, there was no significant difference between the self-evaluation of ‘double deprivation’ farmers and non-deprivation farmers. The probable reason was that most of their families also suffered from some moderate level of deprivation, which was more common in the whole group. Therefore, it was possible to narrow the gap between them and the non-deprived farmers in the evaluation of the vertical sense of gain. For example, in the third class, more than 88% of households also suffered from deprivation of banking and courier services and 66% suffered from deprivation of large medical expenses. As Suppa (2018) asserted, inadequate income was an important factor leading to capacity deprivation, and income poverty as a surrogate indicator could serve as a test of the robustness of the results. However, Table 7 results showed that the relationship between the income poverty category and farmers’ subjective longitudinal sense of gain was not obvious.

Given that the parameter meaning of the ordered probit model is not intuitive, the article also provides the marginal utility estimation result. The results of Table 8 depicted that the gap between the families with severe opportunity deprivation and the families without deprivation was the largest, while the gap between the families with functional deprivation and the families without deprivation was the smallest. However, there was only a significant difference in life satisfaction between double deprivation families and non-deprivation families. At the same time, there was no significant difference between the subjective acquisition of poor families and non-poor families with the threshold of 40% below the median income and between 40% and 50% of the median income. Poor families with extreme income only between 50% and 60% of median income had a lower subjective sense of gain than the non-poor families.

## 6. Discussion

In this study, the survey data from 892 households in six provinces of China are used to explore the associations between the categories of capability deprivation and sense of gain. Unlike previous studies which used the Latent Class Analysis [26], we use a Factor Mixture Model to investigate the group categories of capability deprivation. The advantage of FMM is that it relaxes the assumption of conditional independence and considers the severity of the items. In addition, an ordered probit regression is used to estimate the associations between the categories of capability deprivation and sense of gain. Our results indicate that four deprivation classes are identified and class membership is negatively associated with sense of gain.

First, the nature of the items reflecting the deprivation of function and opportunity is different and the different severity of the items is the reason for the difference of the deprivation mode. According to the factor loading of items, deprivation items have mild, moderate and severe degrees of difference. Specifically, a deprivation item related to a social participation function is less prevalent. On the contrary, deprivation items related to educational opportunities, health functions and participation opportunities are dominant.

Second, capability deprivation is grouped into four modes: double deprivation of opportunity and function, severe deprivation of opportunity, functional deprivation and non-poverty. This result provides more information about structural differences in poverty in the population than the results of the A–F method. What matters most is not the number of deprivations but the types of deprivations [45]. At the same time, inadequate income is one of the causes of disability and the lack of functional needs is the overlap of income poverty and capacity deprivation.

Third, there is a significant correlation between the mode of capability deprivation and sense of gain; although, there is a significant negative relationship between the differences in the patterns of capability deprivation and perceived acquisition. Some social participation function and service opportunity deprivation will lead to a lower perceived acquisition. However, serious deprivation of educational opportunities and social participation opportunities are the main reasons for the lower sense of gain. Inadequate Minsheng public services, including social security, medical and health service, basic social service and public education had a significantly negative effect on sense of gain (Yang, 2018) [52]. In particular, the lack of public resources for the low-income class weakened the sense of gain (Xu et al., 2021) [9].

Our study has several limitations. First, during the evaluation of the FMM model, the inconsistency between fitting indexes may lead to inaccurate classification results (Lubke et al., 2008) [53]. Second, the results are not suitable for causal inference due to the use of cross-sectional data. Third, this study is not comprehensive; it only assesses the overall and relative sense of acquisition. Future studies should, therefore, further explore the impact of capability deprivation on rural households’ sense of longitudinal acquisition.

Considering the above conclusions, this article proposes several suggestions. First, construction of the relative poverty standard of China based on the perspective of function and opportunity. We should include not only functional indicators such as income, material goods, health and education, but also indicators of people’s opportunities to pursue a better life. Second, the target of poverty alleviation should be based on the differences of the groups deprived of ability. In future, we should focus on building an opportunity mechanism for poor groups to enjoy the function of realizing a better life. In particular, full consideration should be given to the needs of farmers in remote areas for better people’s livelihood, such as better educational conditions, more secure pension and reliable old-age services and good community public services. Third, we should enhance farmers’ sense of gain in the process of dealing with relative poverty. We should help the poor to reduce the deprivation of capability; especially eliminating the unequal educational opportunities and social participation opportunities for people in minority areas. They should get better livelihood rights and benefits to enhance their sense of gain.

## 7. Conclusions

This study examined the association between the category of capacity deprivation and the subjective acquisition of rural households. The results show that four deprivation classes are identified and class membership is negatively associated with the sense of gain. Consequently, measures should be taken to help rural households reduce capacity deprivation, meet the needs of a better life and enhance their sense of gain.

## Figures and Tables

**Table 1:** Descriptive statistics of farmers’ sense of acquisition.

Self-Assessment	Life Satisfaction		Self-Confidence		Social Status	
	Frequency	Proportion%	Frequency	Proportion%	Frequency	Proportion%
Very dissatisfied	5	0.56%	7	0.79	8	0.90
Relatively dissatisfied	65	7.34%	41	4.62%	40	4.51
Satisfied	360	40.63%	341	38.44	474	53.44
Relatively satisfied	362	40.86%	410	46.22	293	33.03
Very satisfied	94	10.61%	88	9.92	72	8.12

**Table 2:** Descriptive statistics of deprivation variables.

A Better Life	Activities and Service	Dimensions	Mean Standard	Deviation
Compulsory education	Attend a desirable primary school nearby	Opportunities	0.043	0.202
Compulsory education	Attend a desirable secondary school nearby	Opportunities	0.057	0.232
The social security	Emergency deposit	Functionings	0.210	0.407
The social security	Giving alimony to elders	Functionings	0.096	0.295
The social security	Buy endowment insurance	Functionings	0.089	0.284
Medical and health care	Annual physical examination	Opportunities	0.187	0.390
Medical and health care	Serious illness to urban hospital	Opportunities	0.036	0.186
Medical and health care	Paying for large medical expenses	Opportunities	0.321	0.467
Production and living conditions	Courier service	Opportunities	0.302	0.459
Production and living conditions	Banking services	Opportunities	0.379	0.485
Production and living conditions	Garbage sorting service	Opportunities	0.189	0.392
Cultural activities	Culture to the countryside	Opportunities	0.562	0.496
Cultural activities	Traveling	Opportunities	0.050	0.219
Cultural tradition	Neighbors help each other	Functionings	0.017	0.129
Cultural tradition	Holiday dinners out	Opportunities	0.148	0.355
Cultural tradition	Exchanging gifts and money in daily life	Functionings	0.034	0.180
Cultural tradition	Giving red envelopes for Chinese New Year	Functionings	0.047	0.212

**Table 3:** Factor mixed sub models fitting indexes.

Models	AIC	BIC	a_BIC	Entropy	LMR
Single factor model	7367.963	7487.800	7408.405	–	–
Two-class model	7413.228	7542.651	7456.904	0.777	0.0000
Three-class model	7207.653	7404.185	7273.976	0.811	0.0000
Four-class model	7149.775	7413.416	7238.746	0.838	0.0000
Two class free mean	7301.608	7435.825	7346.903	0.763	0.0245
Three class free mean	7278.358	7422.162	7326.887	0.642	0.2614
Four class free mean	7269.790	7423.181	7321.555	0.631	0.0012
Three class free intercept	7019.244	7278.091	7106.597	0.804	0.0013
Four class free intercept	6979.416	7305.372	7099.416	0.793	0.7797
Three class free intercept and variance	7013.494	7281.928	7104.083	0.630	0.2828
Four class free intercept and variance	6979.493	7319.829	7094.346	0.799	0.0452
Three class free intercept, variance and loading	6990.299	7373.777	7119.712	0.787	0.7648
Four class free intercept, variance and loading	6977.184	7490.085	7150.273	0.868	0.2414

**Table 4:** Intercept estimation results of multiple deprivation category.

Threshold Value	Double Deprivation	Severe Deprivation of Opportunity	Deprivation of Function	Non-Poor
Serious illness to hospital	0.012	0.724	2.050	15.807
Large medical expenses	−0.125	0.646	2.004	2.616
Physical examination	1.274	0.334	1.628	16.383
Giving pension to elders	14.861	0.449	15.972	16.405
Emergency deposit	0.443	0.227	0.215	2.177
Desirable primary school	1.003	−0.251	2.332	3.188
Desirable high school	1.200	0.160	2.366	3.530
Courier service	−1.473	0.004	0.212	0.852
Banking service	−1.410	0.673	−0.099	0.598
Giving red envelopes	−0.370	−0.598	−0.307	1.005
Neighbor help	−0.018	0.331	0.102	1.657
Exchanging gifts and money	0.339	−0.269	−0.026	1.616
Holiday dinners out	0.374	−0.268	0.925	2.587
Proportion (%)	4.60%	5.15%	22.65%	67.60%

**Table 5:** Cross-table of multiple deprivation categories and A–F method results (%).

Classes	K = 0.1	K = 0.2	K = 0.3	K = 0.4	K = 0.5
Double deprivation	9.23	16.47	22.31	34.09	33.33
Severe deprivation of opportunity	10.36	17.67	32.31	59.09	66.67
Deprivation of function	45.50	54.62	45.38	6.82	0.00
Non-poor	34.91	11.24	0.00	0.00	0.00

**Table 6:** Overlap rate of multiple deprivation categories and income poverty (%).

Classes	Per Capita Income (Yuan)	P 40%	P 50%	P 60%
Double deprivation	15,255.88	6.08	5.21	6.00
Severe deprivation of opportunity	15,908.30	8.11	8.33	9.20
Deprivation of function	16,997.91	34.46	34.38	32.40
Non-poor	44,388.11	51.35	52.08	52.40

**Table 7:** Results of ordered probit regression of sense of gain.

Variables	Model 1	Model 2	Model 3	Model 4	Model 5	Model 6
Class-2	−0.7518 *** (0.2211)	−0.5198 ** (0.2079)	−0.6022 *** (0.2129)			
Class-3	−0.5273 *** (0.1834)	−0.2053 (0.1855)	−0.1103 (0.1833)			
Class-4	−0.4886 *** (0.0924)	−0.2367 ** (0.0946)	−0.3167 *** (0.0973)			
Poverty-1				−0.3244 *** (0.1216)	−0.0450 (0.1180)	−0.0443 (0.1191)
Poverty-2				−0.3440 * (0.1837)	−0.1999 (0.1933)	−0.0535 (0.1754)
Poverty-3				−0.5574 *** (0.1521)	−0.3189 ** (0.1569)	−0.4444 *** (0.1628)
Age	0.0088 ** (0.0037)	0.0120 *** (0.0038)	0.0106 *** (0.0038)	0.0087 ** (0.0037)	0.0118 *** (0.0038)	0.0103 *** (0.0038)
Education	0.0270 * (0.0136)	0.0797 *** (0.0138)	0.0566 *** (0.0143)	0.0316 ** (0.0136)	0.0833 *** (0.0139)	0.0623 *** (0.0145)
Marital status	−0.1561 (0.1435)	−0.1126 (0.1612)	−0.2183 (0.1540)	−0.1687 (0.1404)	−0.1238 (0.1625)	−0.2308 (0.1577)
Party status	0.2063 * (0.1263)	0.0979 (0.1277)	0.3335 *** (0.1267)	0.2600 ** (0.1210)	0.1229 (0.1245)	0.3597 *** (0.1243)
Income	0.0636 * (0.0383)	0.0873 ** (0.0371)	0.0404 (0.0325)	0.0406 (0.0409)	0.0923 ** (0.0418)	0.0504 (0.0368)
Business	0.2792 ** (0.1283)	0.2148 (0.1393)	0.2243 * (0.1319)	0.3118 ** (0.1248)	0.2345 * (0.1367)	0.2446 * (0.1294)
Loans	−0.0552 (0.1332)	−0.1473 (0.1571)	0.645 (0.1508)	−0.1351 (0.1315)	−0.1974 (0.1576)	−0.0027 (0.1522)
Livelihood risk	−0.1678 * (0.0945)	−0.1512 (0.0956)	−0.0571 (0.0937)	−0.2614 *** (0.0924)	−0.1937 ** (0.0925)	−0.1937 ** (0.0915)
Land	0.0121 (0.0070)	0.0369 *** (0.0077)	0.0037 (0.0066)	0.0140 ** (0.0070)	0.0388 *** (0.0081)	0.028 (0.0065)
Social capital	0.2122 *** (0.0805)	0.1265 (0.0799)	0.2702 *** (0.0803)	0.2145 *** (0.0806)	0.1255 (0.0795)	0.2718 *** (0.0799)
Central region	−0.0100 (0.0890)	0.1433 (0.0918)	0.2080 ** (0.0927)	0.0589 (0.0883)	0.1210 (0.0925)	0.1786 * (0.0920)
Eastern Region	0.2761 *** (0.0999)	0.3462 *** (0.0981)	0.399 *** (0.0992)	0.2833 *** (0.1000)	0.3521 *** (0.0976)	0.4079 *** (0.0994)
Prob > chi2	0.0000	0.0000	0.0000	0.0000	0.0000	0.0000
Pseudo R2	0.0732	0.0814	0.0738	0.0626	0.0776	0.0685

Note: (1) *, ** and *** represent the significance at the 10%, 5% and 1% level, respectively. (2) Among them, the value corresponds to partial regression coefficient and the robust standard errors are shown in brackets.

**Table 8:** Analysis of the marginal effect of subjective perception of gain.

Sense of Gain	Score	Class-2	Class-3	Class-4	Poverty-1	Poverty-2	Poverty-3
Life Satisfaction	Very dissatisfied	0.015	0.008	0.007 **	0.005 *	0.006	0.012 *
	Relatively dissatisfied	0.110 **	0.068 **	0.062 ***	0.041 **	0.044	0.080 ***
	Satisfied	0.150 ***	0.122 ***	0.115 ***	0.075 ***	0.079 **	0.113 ***
	Relatively satisfied	−0.182 ***	−0.122 ***	−0.112 ***	0.072 **	−0.077 *	−0.132 ***
	Very satisfied	−0.094 ***	−0.076 ***	−0.072 ***	−0.050 ***	−0.052 **	−0.074 ***
Self-confidence	Very dissatisfied	0.013 *	0.004	0.004 *	0.001	0.004	0.008
	Relatively dissatisfied	0.051 *	0.016	0.019 **	0.004	0.018	0.030 *
	Satisfied	0.125 *	0.055	0.063 **	0.012	0.051	0.077 **
	Relatively satisfied	−0.126 **	−0.044	−0.052 **	−0.009	−0.044	−0.074 *
	Very satisfied	−0.064 *	−0.031	−0.035 ***	−0.007	−0.028	−0.042 **
Social status	Very dissatisfied	0.020	0.002	0.008 **	0.001	−0.001	0.016 *
	Relatively dissatisfied	0.059 **	0.008	0.026 ***	0.003	−0.004	0.044 **
	Satisfied	0.125 ***	0.030	0.079 ***	0.011	−0.014	0.090 ***
	Relatively satisfied	−0.142 ***	−0.025	−0.074 ***	−0.010	0.012	−0.104 ***
	Very satisfied	−0.062 ***	−0.015	−0.039 ***	−0.006	0.008	−0.046 ***

Note: *, ** and *** represent the significance at the 10%, 5% and 1% level, respectively.

## Data Availability

The data presented in this study are available on request from the corresponding author.
